# Genome Sequence of “*Candidatus* Walczuchella monophlebidarum” the Flavobacterial Endosymbiont of *Llaveia axin axin* (Hemiptera: Coccoidea: Monophlebidae)

**DOI:** 10.1093/gbe/evu049

**Published:** 2014-03-07

**Authors:** Tania Rosas-Pérez, Mónica Rosenblueth, Reiner Rincón-Rosales, Jaime Mora, Esperanza Martínez-Romero

**Affiliations:** ^1^Centro de Ciencias Genómicas, Universidad Nacional Autónoma de México, Cuernavaca, Morelos, Mexico; ^2^Instituto Tecnológico de Tuxtla Gutiérrez, Tuxtla Gutiérrez, Chiapas, Mexico

**Keywords:** scale insect, γ-Proteobacteria, symbiosis, comparative genomics

## Abstract

Scale insects (Hemiptera: Coccoidae) constitute a very diverse group of sap-feeding insects with a large diversity of symbiotic associations with bacteria. Here, we present the complete genome sequence, metabolic reconstruction, and comparative genomics of the flavobacterial endosymbiont of the giant scale insect *Llaveia axin axin.* The gene repertoire of its 309,299 bp genome was similar to that of other flavobacterial insect endosymbionts though not syntenic. According to its genetic content, essential amino acid biosynthesis is likely to be the flavobacterial endosymbiont's principal contribution to the symbiotic association with its insect host. We also report the presence of a γ-proteobacterial symbiont that may be involved in waste nitrogen recycling and also has amino acid biosynthetic capabilities that may provide metabolic precursors to the flavobacterial endosymbiont. We propose “*Candidatus* Walczuchella monophlebidarum” as the name of the flavobacterial endosymbiont of insects from the Monophlebidae family.

## Introduction

Insects have specialized symbioses with certain bacteria that provide diverse advantages to hosts. Generally, endosymbionts reside inside insect cells, sometimes in unique abdominal structures called bacteriomes (Wernegreen and Wheeler 2009). Endosymbionts have reduced genomes and maintain functions that allow their hosts to live on nutrient-deficient diets such as plant sap or blood (Moya et al. 2008). Endosymbionts from different bacterial phyla have been studied from various sap-sucking insects, mainly aphids and insects in the suborder Auchenorrhyncha (Moran 2007).

Flavobacteria have been found as endosymbionts of several scale insects (Hemiptera: Coccoidea). Until now they have been reported within the families Diaspididae, Lecanodiaspididae, Coccidae, Eriococcidae, Pseudococcidae, Ortheziidae, Coelostomidiidae, and Monophlebidae (Zchori-Fein et al. 2005; Gruwell et al. 2007; Matsuura et al. 2009; Gruwell et al. 2010; Dhami et al. 2012; Rosenblueth et al. 2012). Flavobacteria have been considered to be the primary endosymbionts of scale insects (Gruwell et al. 2007; Rosenblueth et al. 2012). The genome of *Uzinura diaspidicola* (endosymbiont of Diaspididae) was recently sequenced (Sabree et al. 2012). Flavobacteria from scale insects seem to have a single origin, except for *Brownia rhizoecola* (from Pseudococcidae: Phenacoccinae) tribe Rhizoecini (Rosenblueth et al. 2012). Some endosymbiotic Flavobacteria form a clade on the basis of 16S rRNA sequences and have been proposed to cospeciate with their Monophlebidae hosts (Rosenblueth et al. 2012). Besides scale insects, two other Flavobacteria have been reported as insect endosymbionts, *Sulcia muelleri* from Auchenorrhyncha and *Blattabacterium* spp. from basal termites and cockroaches, and their genomes have been fully sequenced (Wu et al. 2006; McCutcheon and Moran 2007; López-Sánchez et al. 2009; McCutcheon et al. 2009; Sabree et al. 2009; McCutcheon and Moran 2010; Woyke et al. 2010; Neef et al. 2011; Huang et al. 2012; Sabree et al. 2012; Patiño-Navarrete et al. 2013; Tokuda et al. 2013). In scale insects, Enterobacteriaceae endosymbionts have been frequently found to coexist with Flavobacteria, including insects from Diaspididae (Rosenblueth et al. 2012); although the genome of *U. diaspidicola* has been sequenced from an insect that only has a single endosymbiont species (Sabree et al. 2012). *Llaveia axin axin* (Monophlebidae) harbors both endosymbionts previously found in scale insects: a flavobacterium and an enterobacterium (Rosenblueth et al. 2012).

*Llaveia axin axin* (Llave) (called “niij” by native people) is a giant scale mainly restricted to tropical lowland regions of the states of Michoacán, Guerrero, and Chiapas in Mexico, and in Guatemala, although there are reports previous to 1995 of its presence in the Mexican states of Guanajuato, Veracruz, and Yucatán (Ben-Dov 2005). It is characterized by its use in the manufacture of native traditional crafts that provide an economic benefit to the local people. A yellow fat that is obtained from the female insect is used to prepare a lacquer to coat traditional art crafts making them resistant to heat, water, and decay. The lacquer can be mixed with other natural products to obtain different colors. It has been used on eating utensils without toxic effects and also as a medicine unguent for external wounds or pain (Williams and MacVean 1995; Martínez 2006).

During its early stages of development, the niij establishes on the young leaves and stems of its host plants, specially *Acacia cochliacantha*, *A. angustissima* (reclassified as *Acaciella angustissima*), *Spondias* sp., and *Jatropha curcas* (Rincón-Rosales and Gutiérrez-Miceli 2008; Suazo-Ortuño et al. 2013) where it sucks the plant sap. As the insect grows, it keeps moving on the plant until it becomes an adult and reaches the principal trunk. Three-year-old plants can have around 300–400 insects but only during a short time in the year (from July to October). Locals collect the females at the end of the rainy season, rendering most of them to obtain the lacquer, but preserving a few to obtain eggs (around 500 eggs per female). Locals place these eggs at the crown of the plants when the rainy season begins and the eggs hatch a few hours later (MacVean 1999). The populations of *L. axin axin* have declined due to its overexploitation, as well as overgrazing, forest fires, and deforestation of the host plants. As they are considered pests of some commercial crops, such as *Spondias purpurea*, they have been eliminated (Rincón-Rosales and Gutiérrez-Miceli 2008; Suazo-Ortuño et al. 2013). Host plant species of *L. axin axin* have in common the production of tannins (Rincón-Rosales and Gutiérrez-Miceli 2008; Islam et al. 2011; de Sousa Araújo et al. 2012) which could be toxic compounds for insects. Endosymbiotic bacteria could have a role in the insect detoxification as in the case of pesticide detoxyfication in stinkbugs by their *Burkholderia* symbiont (Werren 2012). More importantly, monophlebids feed on plant sap, which is a poor source of essential nitrogen compounds (Sandström and Moran 1999).

We present here the complete genome sequence of the *L. axin axin* flavobacterial endosymbiont, a comparative genomic analysis with other flavobacterial endosymbionts, as well as an analysis of its metabolic complementarity with the enterobacterial endosymbiont.

## Materials and Methods

### Nomenclature

In this article we did not use the word “*Candidatus*” as part of the name for symbiotic bacteria, and we italicized the genus and species (e.g., *S. muelleri* instead of *Candidatus* Sulcia muelleri).

### DNA Extraction, PCR, and Cloning

Seven *L. axin axin* female adults were collected from each of the following host plants: *Acaciella angustissima* (Ejido Flores Magón, Mpo. Venustiano Carranza), *Jatropha curcas*, and *Spondias purpurea* (Chiapa de Corzo City) from the state of Chiapas, Mexico. Total DNA from the freshly collected insects was extracted as reported earlier (Rosenblueth et al. 2012). PCR was performed with bacterial universal 16S rRNA primers fD1 (5′-AGAGTTTGATCCTGGCTCAG-3′) and rD1 (5′-AAGGAGGTGATCCAGCC-3′), which amplify products of about 1,500 bases (Weisburg et al. 1991). The PCR products were cloned and 60 individual plasmid clones were sequenced by Macrogen Inc. (Korea). Sequences were compared with the nt database of NCBI using the BlastN algorithm.

### Fluorescent In Situ Hybridization of the Bacteriome

**Fig. 1.—:**
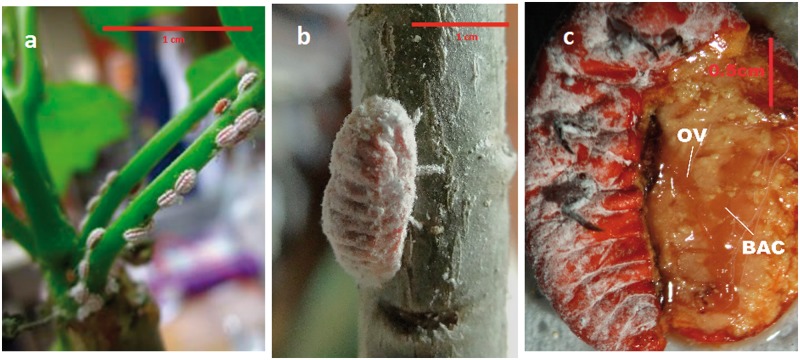
*Llaveia axin axin* insects. (*a*) Twenty-day-old nymphs on *Jatropha curcas* plants, (*b*) adult females on *Jatropha curcas*, (*c*) dissection of left side of abdomen of adult female in a dorsal plane showing the lobed bacteriome (BAC) and ovaries (OV), gut, and malpighian tubules were removed.

Fluorescent in situ hybridization (FISH) was performed as described by Koga et al. (2009) with some modifications. Twenty-day-old freshly collected *L. axin axin* first instar nymphs (fig. 1*a*) were dehydrated with a 30–100% ethanol series, fixed overnight in Carnoy’s solution, washed with ethanol, and treated for a few days in 6% hydrogen peroxide in 80% ethanol. The samples were washed several times with absolute ethanol, then with xylene, and embedded in paraffin. They were cut into 10-μm sections with a rotary microtome and mounted on silane-coated glass slides. Sections were dewaxed through several washes with xylene and ethanol. Hybridization buffer with 100 nM of the probe was added to the samples and was incubated at 28 °C overnight in a humidified chamber. The oligonucleotide probe used was Cy5_DcFlv1450 (5′-Cy5-ATACCTCCGACTTCCAGGA-3′), which targets 16S of Flavobacteria of *Drosicha* sp. (Matsuura et al. 2009) and *L. axin axin*. After washing with PBS, the samples were stained with 2 μg/ml of DAPI and they were mounted with citifluor antifade solution. In order to confirm the specificity of the probe, control experiments were performed with no probe, RNAse digestion, and competitive suppression with excess unlabelled probes. The slides were observed under a Zeiss LSM510 META confocal microscope.

### DNA Preparation and Sequencing

Bacteriomes were dissected in PBS from two frozen (−80 °C) adult females collected in the summer of 2010 in Ejido Flores Magon, Mpo. Venustiano Carranza, Chiapas, Mexico (fig. 1*b* and *c*). DNA from the bacteriomes was purified using the Qiagen Dneasy Blood and Tissue Kit. Six micrograms of the purified DNA was used for Illumina HiSeq 2000 sequencing by the Next Generation Sequencing Division, Macrogen Inc.

For 454 sequencing, 5 μg of DNA were prepared from homogenized and filtered (20 and 11 μm pore size filters) abdomens of ten fresh adult females collected from the same site, using the Qiagen Dneasy Blood and Tissue Kit. Pyrosequencing was carried out in a Roche GS-FLX machine at the Virginia Bioinformatics Institute at Virginia Tech, USA.

### Genome Assembly and Annotation

The Illumina Genome Analyzer System generated 61,593,058 paired-end reads of 100 nt with an insert size of 455 nt. The 454 run generated 78,087 single-end reads with an average length of 190 nt.

Velvet (Zerbino and Birney 2008) was used to make a hybrid assembly with the Illumina and 454 reads generating 118 contigs with an N50 of 231,364. All contigs were compared with the nt database of NCBI using the BlastN algorithm and only two contigs had high-scoring alignments with sequences from Flavobacteria.

A second assembly was run using Phrap (Gordon et al. 1998) taking the velvet contigs as input, generating a single circular contig of 309,299 bp which corresponded to the chromosome sequence of the flavobacterial endosymbiont. Average coverage per nucleotide was 1571.4×. Protein-coding genes were predicted using Glimmer3, GeneMark.hmm, and Blast; tRNAs and a tmRNA were identified with tRNAscan-SE; and rRNAs were identified using the web version of WU-BLAST against the Rfam 11.0 sequence library.

Gene function annotation of the predicted protein-coding genes was based on results of BlastP searches against the RefSeq database and of hidden Markov model searches of the Pfam and TIGRFAM databases. The GenePRIMP pipeline (Pati et al. 2010) was used to search for gene call anomalies and the resulting report was used to perform manual curation of the genome.

### Metabolic Reconstruction

The flavobacterial endosymbiont metabolic pathways were constructed by hand using the Ecocyc and Metacyc databases and the KEGG Automatic Annotation Server assignments as guides.

### Comparative Genomics

Average nucleotide identity between the genomes of *S. muelleri* CARI (from *Clastoptera arizonana*), *Uzinura diaspidicola* ASNER (from *Aspidiotus nerii*), *Blattabacterium* sp. BPLAN (from *Periplaneta americana*), and the flavobacterial endosymbiont of *L. axin axin* was calculated with custom Perl scripts using Mummer alignments output.

Orthologous genes and the core genome of flavobacterial endosymbionts were determined based on BlastP matches between all genes of the four genomes with a high score >75 using the CoreGenes server (Zafar et al. 2002). To show the syntenous blocks of genes between flavobacterial endosymbionts, the genome of the flavobacterial endosymbiont of *L. axin axin* was aligned versus the *S. muelleri*, *U. diaspidicola*, *Blattabacterium* sp., and the free-living pathogen *Flavobacterium psychrophilum* genomes using PROmer (Kurtz et al. 2004) and plotted in figure 5.

### Phylogenetic Analysis

Flavobacteria 16S rRNA sequences from endosymbionts were aligned with ClustalW (Thompson et al. 1994) and manually edited. TIM2 + G model was chosen by FindModel (http://www.hiv.lanl.gov/content/sequence/findmodel/findmodel.html; last accessed March 17, 2014. Posada and Crandall 2001) using Akaike Information Criterion (Akaike 1974). Gaps were not considered and 443 positions were analyzed. Maximum likelihood analysis was performed using PhyML 3.0 (Guindon et al. 2010) with 1,000 bootstrap replicates.

Enterobacteria phylogeny was built by aligning the amino acid sequences of housekeeping genes (*rpoA*, *rpoB*, *rpoD*, *rpoH*, *nusA*, *nusB*, *gyrA*, *pykA*, *dnaE*, and DNA primase) conserved in 20 γ-proteobacteria species including the enterobacterial endosymbiont of *L. axin axin* and other insect endosymbionts with reduced genomes. The alignments were concatenated and all positions containing gaps and missing data were eliminated, leaving a total of 5,565 positions in the final data set. The best model search and phylogenetic analysis were conducted in MEGA5 (Tamura et al. 2011). The evolutionary history was inferred by using the Maximum Likelihood method based on the Whelan and Goldman + Freq. model with 1,000 bootstrap replicates.

GenBank accession numbers from reported sequences used to construct the phylogenies are shown in supplementary table S1, Supplementary Material online.

## Results

### *L. axin axin* Has Two Bacterial Endosymbionts in Bacteriomes

**Fig. 2.—:**
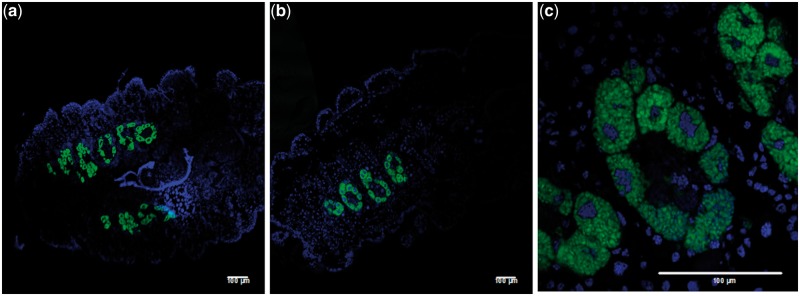
Localization of *Walczuchella monophlebidarum* (green) by fluorescent in situ hybridization in 20-day-old nymphs of *Llaveia axin axin.* (*a*) Dorsal tissue sections of the whole body showing the pair of large lobed bacteriomes where flavobacteria reside, (*b*) sagittal tissue section of the whole body showing one lobed bacteriome, (*c*) enlarged image of a bacteriome lobe.

Only two phylotypes were detected in all the insect female adults sampled, Enterobacteriaceae and Flavobacteria. Figure 1*c* shows one of the two symbiotic organs (bacteriomes) located in the abdominal area of the insect. Figure 2 shows that Flavobacteria are localized in the bacteriomes and that each bacteriome consists of around six lobes. The same type of bacteriome has been found in *Drosicha* spp., which also belongs to Monophlebidae family (Matsuura et al. 2009). Illumina sequences obtained from bacteriome DNA confirmed that the enterobacterial symbiont is also present in the bacteriome. It has also been located in ovary and eggs by PCR.

The maternally inherited endosymbionts of Monophlebidae as well as the two large lobed bacteriomes where they are located had been described earlier by Walczuch (1932) and Tremblay (1989). Monophlebidae has been considered a family of the superfamily Coccoidea in several recent studies (Hodgson and Foldi 2006; Gullan and Cook 2007). Tremblay's (1989) study of the endosymbionts also led him to treat Monophlebidae as a distinct family.

### Proposed Name for Monophlebidae Scale Insects Flavobacterial Endosymbionts

**Fig. 3.—:**
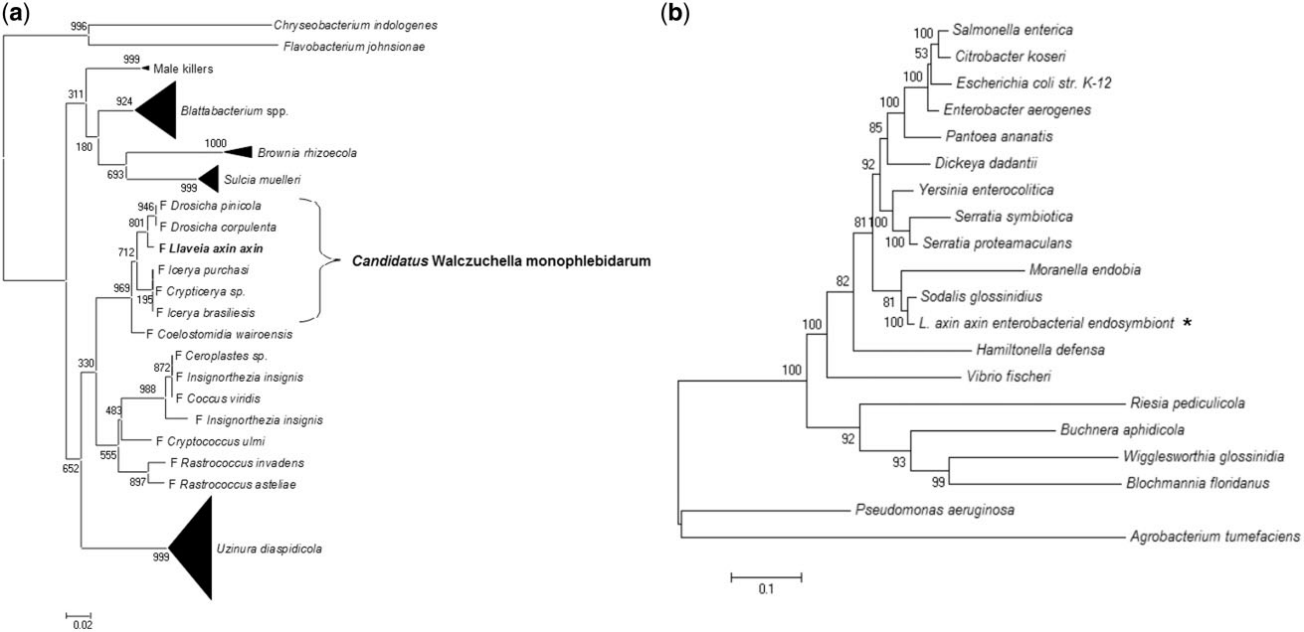
Evolutionary relationships of the flavobacterial and enterobacterial symbionts of *Llaveia axin axin*. (*a*) Phylogeny from flavobacterial endosymbionts inferred from sequences of the 16S rRNA gene. **F** indicates the flavobacterial endosymbiont of the respective insect. Sequences from free-living bacteria were used as outgroups. Bootstrap values for 1,000 replicates are shown adjacent to each node, (*b*) phylogeny from endosymbiotic and free-living enterobacteria inferred from the concatenated amino acid alignment of ten genes using the Maximum Likelihood method based on the Whelan And Goldman + Freq. model. Bootstrap values for 1,000 replicates are shown adjacent to each node. Asterisk indicates the location of the *Llaveia axin axin* enterobacterial symbiont in the tree.

We propose the name “*Candidatus* Walczuchella monophlebidarum” for the flavobacterial endosymbiont living inside bacteriocytes of insects from the family Monophlebidae. The name Walczuchella has been chosen to honor Walczuch (1932) who described the morphology of the bacteriomes in Monophlebidae. *Walczuchella*: Wal.czuch'el.la. N.L. fem. dim. n. *Walczuchella*, named after Walczuch. Previous phylogenetic studies have shown that all the Flavobacteria from Monophlebidae belong to the same clade and suggest that they have cospeciated with their insect hosts in this family (Rosenblueth et al. 2012). That is why we propose the species name to be *monophlebidarum*: mo.no.phle.bi.da'rum N.L. fem. pl. n. Monophlebidae, a zoological family name; N.L. gen. pl. n. *monophlebidarum*, of Monophlebidae. Further analyses should be done to determine whether other Flavobacteria that have been previously obtained from insects of the family Coccidae and Lecanodiaspididae whose 16S rRNA sequences are phylogenetically related to *Walczuchella monophlebidarum* (Rosenblueth et al. 2012) could belong to the same species (fig. 3a).

### General Genomic Features

The genome of *W. monophlebidarum* of *L. axin axin* consists of a circular chromosome of 309,299 bp with an average G + C content of 32.6% and a coding density of 86.2%. It encodes 33 tRNAs corresponding to 20 aminoacids, a single rRNA operon and one tmRNA. There were identified 271 protein-coding sequences (CDSs), 8 of which were classified as hypothetical proteins and the rest had assigned putative biological functions. Twenty-seven CDSs were classified as pseudogenes because of the presence of frameshifts, early stop codons, or because the encoded protein had less than 50% of the length of its closest ortholog in the databases. It is important to note that some of the pseudogenes with frameshifts in homopolymeric tracts may still conserve functionality (Tamas et al. 2008; Wernegreen et al. 2010).

### Metabolic Reconstruction of the Flavobacterial Endosymbiont Genome

**Fig. 4.—:**
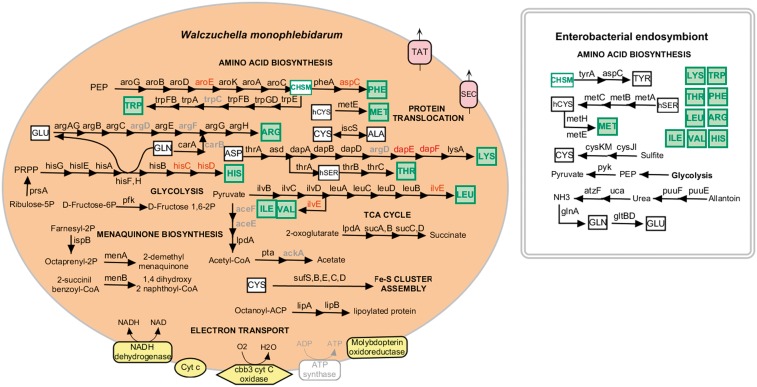
Left: A metabolic reconstruction of *Walczuchella monophlebidarum* of *Llaveia axin axin* based on its genetic content. Right: A representation of the enterobacterial symbiont capabilities for amino acid biosynthesis. Absent genes are shown in red. Pseudogenes are shown in light gray. Green boxes represent essential amino acids. White boxes represent nonessential amino acids.

In figure 4, a reconstruction of *W. monophlebidarum* metabolism is shown. Most genes are involved in protein and amino acids synthesis and RNA processing. *W. monophlebidarum* has a minimal set of genes for genome replication, transcription, and translation. The replication related genes code for DNA polymerase III subunits (α/ε, β, γ/τ, δ, and δ') and DNA gyrase. For transcription, the RNA polymerase core subunits (α, β, and β') are present along with their associated σ70 and σ54 factors. For translation, the complete set of ribosomal proteins is retained along with the three ribosomal RNAs and translation initiation factors (I, II, and III), elongation factors (G, P, Tu, and Ts), and peptide chain release factors (1 and 2).

Almost all of the genes necessary to synthesize the ten essential amino acids are present; however, some of them are annotated as pseudogenes and seven are absent. Biosynthetic pathways for methionine, threonine, tryptophan, and arginine are complete; however, some genes encoding intermediate enzymes in the pathways are annotated as pseudogenes and might not be functional. Pathways for biosynthesis of phenylalanine, histidine, lysine, and branch-chained amino acids are incomplete.

The tRNA synthetases for methionine, asparagine, alanine, and aspartate are missing.

A complete gene set encoding the twin-arginine translocation system (*tatABC*) is present. Genes for an additional protein translocation system were also found. These correspond to the Sec system, which is formed by the *secYEG* operon that encodes a protein conducting channel embedded in the membrane and *secA* that encodes an ATPase that drives translocation. All genes are present except *secG* and spread over the genome not in operons. The gene encoding the auxiliary protein YidC for the biogenesis of both translocation systems is present.

Four subunits of the FOF1 ATP synthase are encoded in the genome. Nonetheless *atpABG* are annotated as pseudogenes and only *atpF* seems to be conserved. However, the flavobacterial genome encodes electron transport proteins: a NADH dehydrogenase, a cbb3-type cytochrome c oxidase complex, and two cytochrome c family proteins, which the flavobacterium can use to produce ATP.

The only genes related to vitamin and cofactor biosynthesis in the genome are *ispB*, *menA*, and *menB* from the menaquinone biosynthetic pathway.

Most of the glycolytic pathway and the TCA cycle are lost except for the pathway converting 2-oxoglutarate to succinate.

As in other insect endosymbionts with reduced genomes, *W. monophlebidarum* lacks genes related to cell envelope biogenesis (fatty acids, phospholipids, and peptidoglycan), DNA recombination, cell motility, defense response, and transporters (McCutcheon and Moran 2012).

The reduced genetic repertoire of the *W. monophlebidarum* genome suggests that it depends on its host or on the secondary symbiont to complement its metabolic and cellular processes.

### Comparative Genomics between Flavobacterial Endosymbionts

*Walczuchella monophlebidarum* from *L. axin axin* shares 85.6% average genomic nucleotide identity with *U. diaspidicola*, 86.8% with *Blattabacterium* sp. BPLAN, and 87.9% with *S. muelleri* CARI, which are the closest bacterial relatives with a sequenced genome*.* Some characteristics of other highly reduced genomes from different insect endosymbionts are shown in table 1.

**Table 1:** Characteristics of Highly Reduced Genomes from Different Insect Endosymbionts

Endosymbiont	Class	Host	Host Diet	Genome Size (bp)^a^	G + C%	CDSs	rRNAs	tRNAs	Pseudo- genes	Coding %
*Flavobacterium psychrophilum*	Flavobacteria	Free living	—	2,861,988	33	2432	18	49	20	85.6
*Walczuchella monophlebidarum*	Flavobacteria	Scale insects	Phloem sap	309,299	32.6	271	3	33	27	86.2
*Uzinura diaspidicola*	Flavobacteria	Scale insects	Parenchyma	263,431	30.2	227	3	30	7	89.3
*Blattabacterium* sp*.* MADAR	Flavobacteria	Termites	Wood	590,336	28	550	3	34	9	94.9
*Blattabacterium* sp*.* BPLAN	Flavobacteria	Cockroaches	Omnivorous	640,442	28	580	3	33	0	94.6
*Sulcia muelleri* CARI	Flavobacteria	Spittlebugs	Sap	276,511	21	246	3	29	0	93.2
*Buchnera aphidicola* APS	Gammaproteobacteria	Aphids	Sap	655,725	26	575	3	32	1	87.5
*Buchnera aphidicola* Cc	Gammaproteobacteria	Aphids	Sap	422,434	20.2	362	3	31	3	86.9
*Carsonella ruddii* PV	Gammaproteobacteria	Psyllids	Phloem sap	159,662	17	182	3	28	0	97.3
*Moranella endobia*	Gammaproteobacteria	Mealybugs	Sap	538,294	43.5	406	5	41	28	79
*Tremblaya princeps* PCIT	Betaproteobacteria	Mealybugs	Sap	138,927	59	121	6	12	2	72.5
*Zinderia insecticola* CARI	Betaproteobacteria	Spittlebugs	Sap	208,564	14	202	3	25	0	92.4
*Hodgkinia cicadicola* Dsem	Alphaproteobacteria	Cicadas	Sap	143,795	58	169	3	15	0	94

^a^Genome size includes chromosome and plasmids if present.

**Fig. 5.—:**
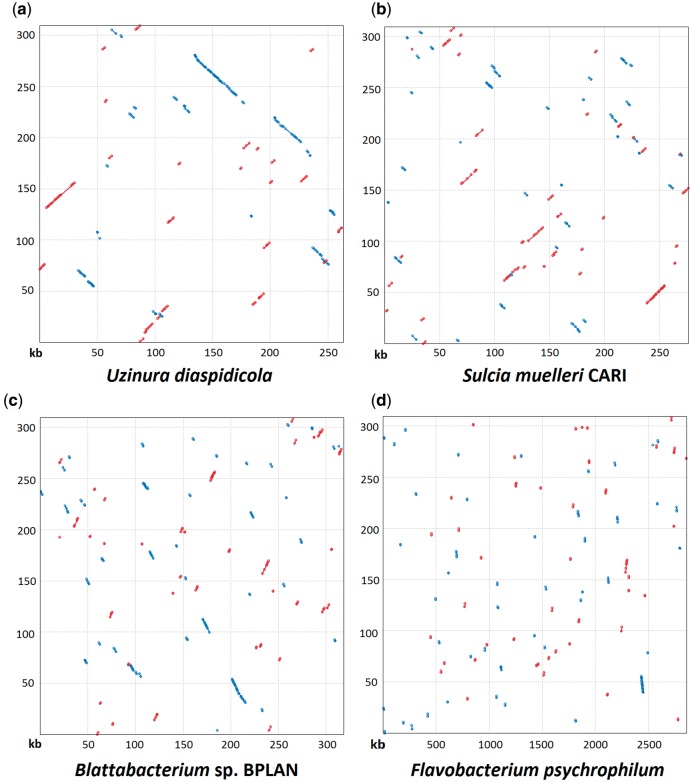
Genomic alignments of *Walczuchella monophlebidarum* of *Llaveia axin axin* (*y* axis) versus (*A*) *Uzinura diaspidicola* (*B*) *Sulcia muelleri* CARI, (*C*) *Blattabacterium* sp. BPLAN, and (*D*) *Flavobacterium psychrophilum*. Forward matches shown in red. Reverse matches shown in blue.

At the genetic level *W. monophlebidarum* shares 209 homologous genes with *U. diaspidicola*, 215 genes with *S. muelleri* and 250 with *Blattabacterium* sp*.* There is functional conservation between these flavobacterial symbionts but no significant genomic synteny. Nevertheless, it can be observed that there is more synteny disruption between *S. muelleri* or *Blattabacterium* sp. and *W. monophlebidarum* than between *U. diaspidicola* and *W. monophlebidarum* (fig. 5). The closer evolutionary relationship between these last two bacteria can be appreciated in the 16S rRNA gene phylogeny of different endosymbiotic Flavobacteria (fig. 3*a*) and has been observed in previously reported phylogenies (Gruwell et al. 2007; Gruwell et al. 2010; Rosenblueth et al. 2012). The core genome of the four endosymbionts corresponds to 157 genes. There are seven genes present in *U. diaspidicola*, *S. muelleri*, and *Blattabacterium* sp. but absent in *W. monophlebidarum*. These genes encode for DNA mismatch repair protein MutL, replicative DNA helicase DnaB, methionyl-tRNA synthetase, two enzymes in the phenylalanine biosynthetic pathway AroE and AspC, malic enzymes MaeA/B, which catalyze the decarboxylation of malate to form pyruvate, and glyceraldehyde-3-phospate dehydrogenase (GapA) required in glycolysis.

Some genes have been lost or pseudogenized in *W. monophlebidarum* but conserved in *S. muelleri* and *U. diaspidicola*, even though these latter two species have more reduced genomes (table 2).

**Table 2:** Genes Retained in the *Sulcia muelleri* and *Uzinura diaspidicola* Genomes That Are Absent in the Genome of *Walczuchella monophlebidarum* of *Llaveia axin axin*

*S. muelleri* Genes	Related Function	*U. diaspidicola* Genes	Related Function
*aroE*	Chorismate biosynthesis	*aroE*	Chorismate biosynthesis
*aspC*	Phenylalanine biosynthesis	*aspC*	Phenylalanine biosynthesis
*aspS*	Aspartyl-tRNA biosynthesis	*dnaB*	Replication
*atpCDEH*	ATP synthase subunits	*eno*	Glycolysis
*dapF*	Lysine biosynthesis pathway	*folC*	Folic acid metabolism
*dnaB*	Replication	*gapA*	Glycolysis
*fabF*	Fatty acids biosynthesis	*glgA*	Glycogen biosynthesis
*fbaA*	Glycolysis	*maeB*	Malate metabolism
*gapA*	Glycolysis	*metG*	Methionyl-tRNA biosynthesis
*ilvE*	val, leu, ile biosynthesis	*mutL*	DNA mismatch repair
*maeA*	Malate metabolism	*pgk*	Glycolysis
*marC*	Integral membrane protein	*queA*	RNA modification
*mutL*	DNA mismatch repair	*rpe*	Pentose degradation
*putA*	Transcription regulator	*sodA*	Oxidative stress response
*ubiE*	Menaquinone biosynthesis	*thiL*	Thiamine biosynthesis
*yaeT*	Outer membrane assembly	*tktC*	Pentose catabolism
primase	Replication	*tktN*	Pentose catabolism
M42 family metaloprotease	Metaloprotease	*trxA*	Electron transfer
*tal*	Transaldolase	Ribokinase	Ribose catabolism
Mrp/Nbp35 ATP-binding protein	ATP-binding	CMP_dCMP deaminase	Deaminase
Signal peptidase	Signal peptidase		
Methyltransferase	Methyltransferase		

Table 3 shows some of the specific differences found in flavobacterial endosymbionts. This comparison showed that *U. diaspidicola* lost all the genes that other flavobacterial symbionts retained related to energy production, menaquinone biosynthesis and protein transport. On the other hand, *U. diaspidicola* has two genes related to vitamin metabolism not found in other flavobacterial symbionts, *folC* for folic acid metabolism and *thiL* for thiamine biosynthesis. Only *Blattabacterium* sp. retained the folate biosynthetic pathway.

**Table 3:** Comparison of Metabolic Components Differentialy Conserved between Flavobacterial Endosymbionts

	*Walczuchella monophlebidarum*	*Uzinura diaspidicola*	*Sulcia muelleri*	*Blattabacterium* sp. BPLAN
Energy production				

ATP synthase	Few subunits	Absent	Present	Present
Cytochrome C	Present	Absent	Present	Present
Cbb3-type cyt C oxydase	Present	Absent	Present	Present
NADH dehydrogenase	Present	Absent	Present	Present

Amino acid biosynthesis				

*ilvE*	Absent	Absent	Present	Present
*dapF*	Absent	Absent	Present	Present
His biosynthesis	Present	Present	Absent	Present
*metE*	Present	Present	Absent	Present
*aspC*	Absent	Present	Present	Present
*argF*	Absent	Present	Present	Present

Vitamins and cofactors				

Menaquinone biosynthesis	Incomplete	Absent	Incomplete	Incomplete
*folC*	Absent	Present	Absent	Present
*thiL*	Absent	Present	Absent	Absent

Protein transport				

Sec system	Present	Absent	Present	Present
Tat system	Present	Absent	Present	Present

Replication and transcription				

*dnaE*	Present	Present	Present	Present
*dnaQ*	Present	Absent	Present	Present
*dnaN*	Present	Absent	Present	Present
*dnaX*	Present	Absent	Absent	Present
*rpoD*	Present	Present	Present	Present
*rpoN*	Present	Present	Absent	Present

Cell envelope				

Fatty acid biosynthesis	Absent	Absent	Only *fabF*	Present
Phospholypids	Absent	Absent	Absent	Present
Peptidoglycans	Absent	Absent	Absent	Present

Carbohydrate metabolism				

Glycolysis	Incomplete	Incomplete	Incomplete	Present
TCA cycle	Incomplete	Incomplete	Incomplete	Present

In general, the set of amino acid biosynthetic genes of *W. monophlebidarum* and *U. diaspidicola* is the same, with the exception of two genes (*aspC* and *argF*) not found in *W. monophlebidarum*. Both *W. monophlebidarum* and *U. diaspidicola* lost *ilvE* which encodes the enzyme for the last step in the branched-chain amino acid biosynthesis pathway while *S. muelleri* and *Blattabacterium* sp. have this gene. Similarly, *S. muelleri* and *Blattabacterium* sp. have *dapF*, a gene from the lysine biosynthetic pathway that is absent in *W. monophlebidarum* and *U. diaspidicola*.

*Walczuchella monophlebidarum*, *U. diaspidicola*, and *Blattabacterium* sp. retained two sigma factors RpoD and RpoN which is unusual in insect endosymbiont genomes.

Only *Blattabacterium* sp. possesses the ability to synthesize the cell envelope components. The glycolytic pathway as well as the TCA cycle are essentially absent in *W. monophlebidarum*, *U. diaspidicola*, and *S. muelleri*.

### Amino Acid Biosynthesis Related Genes in the Enterobacterial Endosymbiont

From the 454 and Illumina sequences, a draft assembly of the enterobacterial endosymbiont genome was obtained consisting of 679 scaffolds with an N50 of 7,713 and an average G + C content of 55.6%. Lengths of scaffolds sum 3.4 Mb of sequence, and taking that as the genome size, we calculate a genome coverage of 34×. We searched for all the essential amino acid biosynthesis genes in the enterobacterial endosymbiont sequences and made a reconstruction of its amino acid biosynthetic pathways. The enterobacterial symbiont has the potential to synthesize the 10 essential amino acids. Only the *aspC* and *argC* genes were not found in the enterobacterial scaffolds. Different from *W. monophlebidarum*, it retains the cobalamin-dependent methionine synthase to synthesize methionine from homocysteine and vitamin B2. The enterobacterial symbiont also has the capacity to synthesize nonessential amino acids. It can produce tyrosine from chorismate and homocysteine from homoserine.

It is also capable of degrading allantoin to urea and then hydrolizing it into CO_2_ and ammonia, which can then be used for glutamine and glutamate production.

It is important to point out that most of the amino acid biosynthesis genes that were absent or pseudogenized in *W. monophlebidarum* genome were present in the enterobacterial symbiont genome.

The size of the enterobacterial symbiont genome is similar to free-living bacteria suggesting a recent change to a symbiotic lifestyle, as in its close relative *Sodalis glossinidius* (Toh et al. 2006).

## Discussion

The genetic content of *W. monophlebidarum* genome suggests a role in synthesizing essential amino acids for the host. According to its metabolic reconstruction, the flavobacterium needs to be provided with some precursors for the amino acid biosynthesis like PEP for phenylalanine and tryptophan production, ribulose-5P for histidine synthesis, pyruvate for branched-chain amino acid production, and some non-essential amino acids for arginine, methionine, lysine, and threonine synthesis (fig. 4).

The enterobacterial symbiont could be supplying most of these precursors as it has the potential to make ribulose-5P from ribose-1P and also PEP and pyruvate from glycolysis. It is also capable of producing homocysteine from homoserine for methionine biosynthesis.

Interestingly, the enterobacterial symbiont could recycle the waste nitrogen from the insect in the form of allantoin to provide precursors for amino acid biosynthesis. Nitrogen recycling potential has also been reported in *Blattabacterium* sp. but through a different pathway. It is possible that *W. monophlebidarum* could assimilate distinct nitrogen products as it retains RpoN, a nitrogen-related gene regulator.

The metabolic precursor production by the enterobacterial symbiont suggests metabolic complementarities between the two endosymbionts. However, their genetic content for essential amino acid biosynthesis overlaps. This might mean that 1) both endosymbionts supply the insect host with essential amino acids or that 2) because of the loss and degradation of many essential genes for viability and amino acid biosynthesis in *W. monophlebidarum*, it has become incapable of fulfilling its role in the symbiotic relationship with the insect, and the enterobacterial symbiont now fulfills the flavobacterium's former functions.

As with *Sodalis glossinidius* and *Wigglesworthia glossinidia* (Snyder et al. 2010), the primary endosymbiont may have the capacity to produce a nutrient that the secondary symbiont needs and is not capable of producing, in this case thiamine. This could lead to the stable coexistence of both endosymbionts.

*Walczuchella monophlebidarum* and *U. diaspidicola* have essentially the same genetic potential for amino acid biosynthesis, the main difference being the presence of frameshifts in some genes of these pathways of *W. monophlebidarum*. *Sulcia muelleri* differs from them because it has a secondary symbiont capable of synthesizing methionine and histidine, capabilities that have been lost in *S. muelleri*.

Perhaps the more degraded state of metabolic pathways of *W. monophlebidarum* in comparison with those of *U. diaspidicola*, which has a similar host and environment, can be explained by the presence of the enterobacterial symbiont in *L. axin axin*, as this reduces the selection forces on the flavobacterium genes. On the other hand, it is interesting how *S. muelleri*, with a similar life style in an insect host with a similar diet and also accompanied by a secondary endosymbiont as in the case of the *L. axin axin* flavobacterium, has maintained amino acid biosynthetic pathway’s integrity.

The high number of pseudogenes in the *W. monophlebidarum* genome compared with other reduced genomes suggests that it is still under a genomic reduction process. Despite the lack of genomic synteny observed in the flavobacteria of sap-feeding insects, they have a similar genome size and a significant functional convergence.

## Supplementary Material

Supplementary table S1 is available at *Genome Biology and Evolution* online (http://www.gbe.oxfordjournals.org/).

Supplementary Data

## References

[evu049-B1] Akaike H (1974). A new look at the statistical model identification. IEEE Trans Autom Control..

[evu049-B2] Ben-Dov Y (2005). A systematic catalogue of the scale insect family Margarodidae (Hemiptera: Coccoidea) of the world.

[evu049-B53] de Sousa Araújo TA, de Almeida e Castro VT, de Amorim EL, de Albuquerque UP (2012). Habitat influence on antioxidant activity andtannin concentrations of Spondias tuberosa. Pharm Biol..

[evu049-B3] Dhami MK, Turner AP, Deines P, Beggs JR, Taylor MW (2012). Ultrastructural and molecular characterization of a bacterial symbiosis in the ecologically important scale insect family Coelostomidiidae. FEMS Microbiol Ecol..

[evu049-B4] Gordon D, Abajian C, Green P (1998). Consed: a graphical tool for sequence finishing. Genome Res..

[evu049-B5] Gruwell ME, Hardy NB, Gullan PJ, Dittmar K (2010). Evolutionary relationships among primary endosymbionts of the mealybug subfamily Phenacoccinae (Hemiptera: Coccoidea: Pseudococcidae). Appl Environ Microbiol..

[evu049-B6] Gruwell ME, Morse GE, Normark BB (2007). Phylogenetic congruence of armored scale insects (Hemiptera: Diaspididae) and their primary endosymbionts from the phylum Bacteroidetes. Mol Phylogenet Evol..

[evu049-B7] Guindon S (2010). New algorithms and methods to estimate maximum-likelihood phylogenies: assessing the performance of PhyML 3.0. Syst Biol..

[evu049-B8] Gullan PJ, Cook LG (2007). Phylogeny and higher classification of the scale insects (Hemiptera: Stenorrhyncha: Coccoidea). Zootaxa.

[evu049-B9] Hodgson CJ, Foldi I (2006). A review of the Margarodidae *sensu* Morrison (Hemiptera: Coccoidea) and some related taxa based on the morphology of adult males. Zootaxa.

[evu049-B10] Huang CY, Sabree ZL, Moran NA (2012). Genome sequence of *Blattabacterium* sp. strain BGIGA, endosymbiont of the *Blaberus giganteus* cockroach. J Bacteriol..

[evu049-B52] Islam AKMA, Yaakob Z, Anuar N (2011). Jatropha: A multipurpose plant with considerable potential for the tropics. Sci Res Essays..

[evu049-B11] Koga R, Tsuchida T, Fukatsu T (2009). Quenching autofluorescence of insect tissues for in situ detection of endosymbionts. Appl Entomol Zool..

[evu049-B12] Kurtz S (2004). Versatile and open software for comparing large genomes. Genome Biol..

[evu049-B13] López-Sánchez MJ (2009). Evolutionary convergence and nitrogen metabolism in *Blattabacterium* strain Bge, primary endosymbiont of the cockroach *Blattella germanica*. PLoS Genet..

[evu049-B14] MacVean CM (1999). Laca artesanal producto del insecto niij. Revista Galería Guatemala (Guatemala, Fundación G&T).

[evu049-B15] Martínez FM (2006). La laca de los Achíes. Artículo de semanario de prensa libre. http://servicios.prensalibre.com/pl/domingo/archivo/revistad/2006/marzo06/190306/dfondo.shtml.

[evu049-B16] Matsuura Y (2009). Huge symbiotic organs in giant scale insects of the genus *Drosicha* (Coccoidea: Monophlebidae) harbor flavobacterial and enterobacterial endosymbionts. Zoolog Sci..

[evu049-B17] McCutcheon JP, Moran NA (2007). Parallel genomic evolution and metabolic interdependence in an ancient symbiosis. Proc Natl Acad Sci U S A..

[evu049-B18] McCutcheon JP, Moran NA (2010). Functional convergence in reduced genomes of bacterial symbionts spanning 200 My of evolution. Genome Biol Evol..

[evu049-B19] McCutcheon JP, Moran NA (2012). Extreme genome reduction in symbiotic bacteria. Nat Rev Microbiol..

[evu049-B20] McCutcheon JP, McDonald BR, Moran NA (2009). Convergent evolution of metabolic roles in bacterial co-symbionts of insects. Proc Natl Acad Sci U S A..

[evu049-B21] Moran NA (2007). Symbiosis as an adaptive process and source of phenotypic complexity. Proc Natl Acad Sci U S A..

[evu049-B22] Moya A, Peretó J, Gil R, Latorre A (2008). Learning how to live together: genomic insights into prokaryote-animal symbioses. Nat Rev Genet..

[evu049-B23] Neef A (2011). Genome economization in the endosymbiont of the wood roach *Cryptocercus punctulatus* due to drastic loss of amino acid synthesis capabilities. Genome Biol Evol..

[evu049-B24] Pati A (2010). GenePRIMP: a gene prediction improvement pipeline for prokaryotic genomes. Nat Methods..

[evu049-B25] Patiño-Navarrete R, Moya A, Latorre A, Peretó J (2013). Comparative genomics of *Blattabacterium cuenoti*: the frozen legacy of an ancient endosymbiont genome. Genome Biol Evol..

[evu049-B26] Posada D, Crandall KA (2001). Selecting the best-fit model of nucleotide substitution. Syst Biol..

[evu049-B27] Rincón-Rosales R, Gutiérrez-Miceli FA (2008). Biological characteristics of *Acaciella angustissima* (Mill.) Britton & Rose in natural habitat and assessment o its bark potential in Chiapas, Mexico. Agrociencia.

[evu049-B28] Rosenblueth M, Sayavedra L, Sámano-Sánchez H, Roth A, Martínez-Romero E (2012). Evolutionary relationships of flavobacterial and enterobacterial endosymbionts with their scale insect hosts (Hemiptera: Coccoidea). J Evol Biol..

[evu049-B29] Sabree ZL, Huang CY, Okusu A, Moran NA, Normark BB (2012). The nutrient supplying capabilities of *Uzinura*, an endosymbiont of armored scale insects. Environ Microbiol..

[evu049-B30] Sabree ZL, Kambhampati S, Moran NA (2009). Nitrogen recycling and nutritional provisioning by *Blattabacterium*, the cockroach endosymbiont. Proc Natl Acad Sci U S A..

[evu049-B31] Sabree ZL (2012). Genome shrinkage and loss of nutrient-providing potential in the obligate symbiont of the primitive termite Mastotermes darwiniensis. Appl Environ Microbiol..

[evu049-B32] Sandström J, Moran N (1999). How nutritionally imbalanced is phloem sap for aphids-?. Entomol Exp Appl..

[evu049-B33] Snyder AK, Deberry JW, Runyen-Janecky L, Rio RV (2010). Nutrient provisioning facilitates homeostasis between tsetse fly (Diptera: Glossinidae) symbionts. Proc Biol Sci..

[evu049-B34] Suazo-Ortuño I, del Val-De Gortari E, Benítez-Malvido J (2013). Rediscovering an extraordinary vanishing bug: *Llaveia axin axin*. Revista Mexicana de Biodiversidad.

[evu049-B35] Tamas I (2008). Endosymbiont gene functions impaired and rescued by polymerase infidelity at poly (A) tracts. Proc Natl Acad Sci U S A..

[evu049-B36] Tamura K (2011). MEGA5: molecular evolutionary genetics analysis using maximum likelihood, evolutionary distance and maximum parsimony methods. Mol Biol Evol..

[evu049-B37] Thompson JD, Higgins DG, Gibson TJ (1994). Clustal W: improving the sensitivity of progressive multiple sequence alignment through sequence weighting, position-specific gap penalties and weight matrix choice. Nucleic Acids Res..

[evu049-B38] Toh H (2006). Massive genome erosion and functional adaptations provide insights into the symbiotic lifestyle of *Sodalis glossinidius* in the tsetse host. Genome Res..

[evu049-B39] Tokuda G (2013). Maintenance of essential amino acid synthesis pathways in the *Blattabacterium cuenoti* symbiont of a wood-feeding cockroach. Biol Lett..

[evu049-B40] Tremblay E, Schwemmler W, Gassner G (1989). Coccoidea endocytobiosis. Insect endocytobiosis: morphology, physiology, genetics, evolution.

[evu049-B41] Walczuch A (1932). Studien an Coccidensymbionten. Z Morphol Ökol Tiere.

[evu049-B42] Weisburg WG, Barns SM, Pelletier DA, Lane DJ (1991). 16S ribosomal DNA amplification for phylogenetic study. J Bacteriol..

[evu049-B43] Wernegreen JJ, Kauppinen SN, Degnan PH (2010). Slip into something more functional: selection maintains ancient frameshifts in homopolymeric sequences. Mol Biol Evol..

[evu049-B44] Wernegreen JJ, Wheeler DE (2009). Remaining flexible in old alliances: functional plasticity in constrained mutualisms. DNA Cell Biol..

[evu049-B45] Werren JH (2012). Symbionts provide pesticide detoxification. Proc Natl Acad Sci U S A..

[evu049-B46] Williams M, MacVean CM (1995). Ethnococcidology: use of the giant margarodids, *Llaveia* spp. (Homoptera: Coccoidea: Margarodidae), by indigenous peoples of Mesoamerica in their culture, medicine and arts. Israel J Entomol..

[evu049-B47] Woyke T (2010). One bacterial cell, one complete genome. PLoS One.

[evu049-B48] Wu D (2006). Metabolic complementarity and genomics of the dual bacterial symbiosis of sharpshooters. PLoS Biol..

[evu049-B49] Zafar N, Mazumder R, Seto D (2002). CoreGenes: a computational tool for identifying and cataloging “core” genes in a set of small genomes. BMC Bioinformatics.

[evu049-B50] Zchori-Fein E, Ben-Dov Y, Portnoy V, Katzir N, Erkılıc LB, Kaydan MB (2005). Distribution of the endosymbiont *Cardinium hertigii* in scale insects (Hemiptera: Coccoidea). Proceedings of the Tenth International Symposium on Scale Insect Studies; 2004 April 19–23.

[evu049-B51] Zerbino DR, Birney E (2008). Velvet: algorithms for de novo short read assembly using de Bruijn graphs. Genome Res..

