# Supplementary material for: Genome Sequence of “Candidatus Walczuchella monophlebidarum” the Flavobacterial Endosymbiont of Llaveia axin axin (Hemiptera: Coccoidea: Monophlebidae)
Source: Genome Biol Evol. 2014 Mar 7;6(3):714–26. doi: 10.1093/gbe/evu049 (PMC3971599; doi:10.1093/gbe/evu049)
Supplement: Supplementary Data [file supp_6_3_714__index.html]

Genome sequence of “Candidatus Walczuchella monophlebidarum” the flavobacterial endosymbiont of Llaveia axin axin (Hemiptera: Coccoidea: Monophlebidae) — Genome Sequence of “Candidatus Walczuchella monophlebidarum” the Flavobacterial Endosymbiont of Llaveia axin axin (Hemiptera: Coccoidea: Monophlebidae) — Supplementary Data 

# Genome Sequence of “*Candidatus* Walczuchella monophlebidarum” the Flavobacterial Endosymbiont of *Llaveia axin axin* (Hemiptera: Coccoidea: Monophlebidae)

## Supplementary Data

files

**Files in this Data Supplement:**

- Supplementary Data - docx file
