# Supplementary material for: Genome Sequence of “Candidatus Walczuchella monophlebidarum” the Flavobacterial Endosymbiont of Llaveia axin axin (Hemiptera: Coccoidea: Monophlebidae)
Source: Genome Biol Evol. 2014 Mar 7;6(3):714–26. doi: 10.1093/gbe/evu049 (PMC3971599; doi:10.1093/gbe/evu049)
Supplement: Supplementary Data [file supp_evu049_Rosas-Perez_etal_Suppl_Table1.docx]

**Table S1**

GenBank accession numbers used to construct the phylogenetic trees.

| **Species or clade** | **GenBank numbers** |
| --- | --- |
| *Chryseobacterium indologenes* | AM232813 |
| *Flavobacterium johnsionae* | EU239171 |
| Male killers clade | Y13889, AJ009687, FN557165 |
| *Blattabacterium* spp. clade | AF322469, AF363710, AF322468, AF322459, NR_102785, AF322461, AF322462, AF363703, AY631417, AB211183, AY631413, AB211175, AB211184, AF310161, NR_102962, AB231592, AB231588, AB231590, AB231601, AB231602, AB231593, AB231608, AB231609, AB231607, NR_102963, X75622, X75626, AM182899, NR 102498, NR_102965, AJ514943, AJ514944, AJ514945 |
| *Brownia rhizoecola* clade | HM449968, HM449967, HM449971, HM449970, HM449969, HM449966, HM449965 |
| *Sulcia muelleri* clade | DQ066631, AB772240, AB772241, DQ066633, AB772239, AB772236, AB772244, AB772247, AB772248, AB772246, DQ066630, AB772245, CP000770, CP002163, DQ066634, AB772258 |
| Flavobacteria from *Drosicha pinicola* | AB491199 |
| Flavobacteria from *Drosicha corpulenta* | AB491197 |
| Flavobacteria from *Llaveia axin axin* | HQ893826 |
| Flavobacteria from *Icerya purchasi* | DQ133550 |
| Flavobacteria from *Crypticerya* sp. | HQ893828 |
| Flavobacteria from *Icerya brasiliensis* | DQ133551 |
| Flavobacteria from *Coelostomidia wairoensis* | JQ322766 |
| Flavobacteria from *Ceroplastes* sp. | HQ893829 |
| Flavobacteria from *Insignorthezia insignis* | HQ893824 |
| Flavobacteria from *Coccus viridis* | HQ893830 |
| Flavobacteria from *Insignorthezia insignis* | HQ893825 |
| Flavobacteria from *Cryptococcus ulmi* | DQ133549 |
| Flavobacteria from *Rastrococcus invadens* | HM449963 |
| Flavobacteria from *Rastrococcus asteliae* | HM449962 |
| *Uzinura diaspidicola* clade | DQ868843, DQ868841, HQ893833, DQ868795, DQ868803, HQ893832, DQ868801, DQ868799, GQ424856, DQ133561, DQ868857, DQ868812, DQ868811, DQ133560, GQ424840, DQ868796, DQ868798, DQ868820, GQ424913, DQ868810, GQ424835, GQ424912, GQ424887, GQ424843, DQ893834, DQ868814, DQ868813, DQ868797, GQ424943, GQ424842, DQ133556, NR_102496, GQ424877, GQ424838, GQ424901, DQ868800, GQ424902, GQ424940, GQ424952, DQ133557, DQ868835, DQ868838, GQ424850, DQ868844, GQ424928, GQ424924, DQ868834, GQ424860, GQ424862, GQ424927, GQ424922 |
| *Yersinia enterocolitica* subsp. enterocolitica 8081 | YP_001007443, YP_001007838, YP_001005708, YP_001004803, YP_001007340, YP_001006604, YP_001008043, YP_001004668, YP_001007839, YP_001004614 |
| *Wigglesworthia glossinidia* endosymbiont of Glossina morsitans morsitans (Yale colony) | YP_005267442, YP_005267350, YP_005267375, YP_005267596, YP_005267356, YP_005267683, YP_005267251, YP_005267298, YP_005267351, YP_005267159 |
| *Vibrio fischeri* ES114 | YP_205330, YP_205635, YP_204587, YP_203868, YP_204087, YP_206452, YP_203645, YP_205797, YP_205637, YP_205833 |
| *Serratia symbiotica* str. Cinara cedri | YP_005060402, YP_005060730, YP_005060617, YP_005060257, YP_005060627, YP_005060551, YP_005060811, YP_005060840, YP_005060729, YP_005060824 |
| *Serratia proteamaculans* 568 | YP_001479999, YP_001480521, YP_001479495, YP_001476724, YP_001477306, YP_001478416, YP_001480741, YP_001476515, YP_001480522, YP_001476462 |
| *Sodalis glossinidius* str. morsitans | YP_455608, YP_453932, YP_455262, YP_454056, YP_454333, YP_455118, YP_455933, YP_453814, YP_453931, YP_453767 |
| *Salmonella enterica* subsp. enterica serovar Typhi str. CT18 | NP_454838, NP_457601, NP_456817, NP_457669, NP_455012, NP_456455, NP_458485, NP_457917, NP_457602, NP_458353 |
| *Riesia pediculicola* USDA | YP_003603208, YP_003603422, YP_003602956, YP_003603137, YP_003603054, YP_003603010, YP_003603281, YP_003603263, YP_003603421, YP_003602903 |
| *Pseudomonas aeruginosa* PAO1 | NP_252330, NP_249268, NP_251858, NP_253433, NP_252741, NP_253019, NP_252928, NP_252960, NP_249267, NP_249067 |
| *Pantoea ananatis* LMG 20103 | YP_003519103, YP_003521684, YP_003520907, YP_003518751, YP_003519266, YP_003520488, YP_003521892, YP_003518510, YP_003521685, YP_003522017 |
| *Moranella endobia* PCIT | YP_004706838, YP_004706745, YP_004706936, YP_004706807, YP_004706667, YP_004706657, YP_004706908, YP_004706921, YP_004706744, YP_004706761 |
| *Hamiltonella defensa* 5AT | YP_002923125, YP_002923533, YP_002924287, YP_002923242, YP_002924665, YP_002923677, YP_002924584, YP_002923592, YP_002923534, YP_002924820 |
| *Enterobacter aerogenes* KCTC 2190 | YP_004592536, YP_004590949, YP_004595018, YP_004591064, YP_004592703, YP_004593623, YP_004591172, YP_004591828, YP_004590950, YP_004591310 |
| *Escherichia coli* str. K-12 substr. MG1655 | NP_414726, NP_417538, NP_416734, NP_417638, NP_414950, NP_416191, NP_417754, NP_418414, NP_417539, NP_417918 |
| *Dickeya dadantii* 3937 | YP_003881851, YP_003881377, YP_003881998, YP_003881400, YP_003881918, YP_003882913, YP_003884830, YP_003880995, YP_003881376, YP_003880910 |
| *Citrobacter koseri* ATCC BAA-895 | YP_001454703, YP_001455957, YP_001452135, YP_001456059, YP_001454287, YP_001453275, ABV15753, YP_001454538, YP_001455958, YP_001456366 |
| *Buchnera aphidicola* str. APS | NP_240067, NP_239893, NP_240011, NP_240196, NP_240274, NP_240141, NP_240306, NP_239875, NP_239892, NP_239866 |
| *Blochmannia floridanus* | NP_878583, NP_878369, NP_878759, NP_878410, NP_878533, NP_878736, NP_878515, NP_878832, NP_878368, NP_878894 |
| *Agrobacterium tumefaciens* | WP_012651238, WP_020812207, WP_003525064, WP_003507172, WP_003502227, WP_020808592, AAD47422, WP_003523727, WP_020812239, P_003522581 |
|  |  |
